# Balancing efficacy and quality of life measurements among metastatic renal cell carcinoma (RCC) studies

**DOI:** 10.18632/oncoscience.528

**Published:** 2021-03-21

**Authors:** Jeanny B. Aragon-Ching

**Affiliations:** ^1^GU Medical Oncology, Inova Schar Cancer Institute, Fairfax, VA, USA

**Keywords:** mRCC, VEGF-TKI, Q-TWiST, CABOSUN trial

## Abstract

Metastatic renal cell carcinoma (mRCC) treatments have rapidly evolved in the last few years. While vascular endothelial growth factor (VEGF) inhibition had previously been the mainstay of treatment for first-line advanced RCC therapy in the past decade, it has now rapidly changed into combination checkpoint inhibitors with or without VEGF TKIs, although there remains a role for VEGF tyrosine kinase inhibitor monotherapy for patients with favorable-risk disease and for those with intermediate and poor-risk disease with the use of cabozantinib. Perspectives on the Quality-adjusted survival Time without Symptoms of disease or Toxicity (Q-TWiST) analysis for the CABOSUN trial, as well as different aspects of efficacy regarding different first-line therapy for advanced or metastatic RCC are discussed herein.

Treatment of metastatic RCC has been rapidly evolving over the past few years. While the use of VEGF TKIs (vascular endothelial growth factors – tyrosine kinase inhibitors) had been the cornerstone of treatment for advanced or metastatic renal cell carcinomas (RCC) since the approval of sunitinib and pazopanib [1], it soon got supplanted by the advent of treatment with the use of immune-oncology (IO) drugs. The first line treatment using checkpoint inhibitor (CPI) drugs or IO drugs versus the combination of IO with VEGF TKIs are rapidly evolving. In addition, the second-line treatment with nivolumab monotherapy or VEGF-TKI treatment with cabozantinib, lenvatinib with everolimus, axitinib, have all shaped the landscape of treatment for mRCC.

The first line therapy with mRCC spans the use of IO/IO combination with Checkmate 214 using nivolumab and ipilimumab [2, 3], the IO/VEGF-TKI combination which are plentiful including the earliest combination with atezolizumab with bevacizumab in the IMmotion 151 trial [4], Keynote 426 which combines pembrolizumab and axitinib [5], Javelin Renal 101 using avelumab and axitinib [6], Checkmate 9ER with nivolumab and cabozantinib [7], as well as VEGF-TKI monotherapy with cabozantinib based on the CABOSUN trial [8], all with varying regulatory approval (see Table 1). On the other hand, newer studies including CLEAR [9], that evaluated pembrolizumab and lenvatinib, all have shown benefit over sunitinib alone. These first line trials assessed and evaluated different primary endpoints and have varying overall response rates but also different quality of life results. While overall survival has historically been used as the most important parameter of benefit, some of the combination therapies or monotherapy have been approved based on progression-free survival (PFS) alone. Therefore, choosing the most optimal patient population for specific types of therapy are also poorly defined.

Given the abundance of first-line studies available, all of which showing advantages in the primary endpoint that they were designed for and almost unanimously superior to sunitinib alone, it is increasingly difficult to determine which combination or treatment is ultimately suitable for which patients at the appropriate time in their disease course. Certainly, one important aspect of importance to evaluate is the quality of life. While toxicities in general are different with checkpoint inhibitor use versus VEGF TKI use, the combination brings about potential overlap in the adverse effects with the use of both these agents. While cross-comparison amongst different trials is not recommended, there are also important considerations regarding toxicity profile that would lead to better tolerability in the choice of different therapies.

**Table 1:** Table 1: Selected first-line trials for advanced or metastatic renal cell carcinoma (mRCC)

Trial	Phase of trial/MOA	Doses and arms of therapy (n)	Primary Endpoints	Endpoints/ Responses	Comments
CABOSUN	Phase II/ TKI	Cabozantinib 60 mg vs. sunitinib 50 mg daily (4 weeks on/2 weeks off) (n = 157)	PFS	PFS: C=8.2 mos vs. S = 5.6 mos; ORR: C = 33% vs. S = 12%	FDA-approved December 19, 2017 for first-line advanced RCC
IMmotion 151	Phase III/ PD-L1 + TKI	Atezolizumab 1200 mg IV + bevacizumab 15 mg/kg IV q 3 weeks (6-week cycle) vs. sunitinib 50 mg daily (4 weeks on/2 weeks off) (n = 915)	PFS in PD-L1+; OS in ITT	PFS in PD-L1: Atezo + Bev = 11.2 mos vs S = 7.7 mos; P = 0.02; OS: Atezo + Bev = NR vs S =23.3 mos	Not FDA-approved
Checkmate 214	Phase III/ PD-L1 and CTLA-4 inhibitor	Nivolumab IV + Ipilimumab 1 mg/kg x4 q 3 weeks then Nivolumab q 2 weeks vs. sunitinib 50 mg daily (n =1096)	OS, ORR and PFS in intermediate and poor-risk	N/I: 18-mo OS = 75%; mOS=NR; ORR = 42% vs. S: 18-mo OS=60%; mOS= 26 mos; ORR=27%;	FDA approved on April 16, 2018 for intermediate and poor-risk
JAVELIN Renal 101	Phase III/ PD-L1 + TKI	Avelumab 10 mg/kg IV q2 weeks + Axitinib 5mg BID (6-week cycle) vs. sunitinib 50 mg daily (4 weeks on/2 weeks off) (n = 886)	PFS, OS in PD-L1+	mPFS: Ave + axi = 13.8 mos vs. S = 7.2 mos; ORR: Ave + axi = 55.2% vs. S = 25.5%;	FDA approved on May 14, 2019 for front-line treatment advanced RCC
Checkmate 9ER	Phase III/ PD-L1 + VEGF TKI	Nivolumab 240 mg IV q 2 weeks + cabozantinib 40 mg po vs. sunitinib 50 mg daily (4 weeks on/2 weeks off) (n=	PFS as determined by BICR	mPFS N/C = 16.6 mos vs S = 8.3 mos	FDA approved on January 20, 2021 for advanced RCC
KEYNOTE-426	Phase III/ PD-1 + TKI	Pembrolizumab 200mg IV q 3 weeks + axitinib 5mg BID (6-week cycle) vs. sunitinib 50mg daily (4 weeks on/2 weeks off) (n = 840)	PFS, OS	mPFS: Pem + axi = 15.1 mos vs. S = 11.1 mos; ORR: Pem + axi =59.3% vs. S = 35.7%; P<0.001	FDA approved on April 19, 2019 for first-line treatment advanced RCC
KEYNOTE-581/ CLEAR	Phase III/ PD-1 + TKI	Pembrolizumab 200mg IV q 3 weeks + lenvatinib 20 mg/day vs. everolimus 5 mg/day + lenvatinib 18 mg/day vs. sunitinib 50mg daily (4 weeks on/2 weeks off) (n = 735)	PFS	PFS: Len + P = 23.9 mos vs S = 9.2 mos; Len + eve = 14.7 mos vs. S = 9.2 mos; OS = Len + P vs S; HR = 0.66;	Not FDA approved yet

VEGF = vascular endothelial growth factor; TKI = tyrosine kinase inhibitor; PD-1 = programmed cell death protein 1; PD-L1 – programmed death-ligand 1; n = number; q = every; BID = twice a day; ORR = objective response rate; OS = Overall survival; PFS = progression-free survival; mPFS = median PFS; mOS = median OS; ITT = intention-to-treat population; mos = months; AE = adverse event; DCR = disease control rate; Ave = avelumab; Axi = axitinib; BICR = Blinded independent central review; S = sunitinib; N = Nivolumab; I = ipilimumab; C = cabozantinib; Len = Lenvatinib; Pem = pembrolizumab; eve = everolimus; Atezo = atezolizumab;.

The quality of life analyses of the CABOSUN trial [10] evaluated the Q-TWiST (Quality-adjusted survival Time without Symptoms of disease or Toxicity) also demonstrated the quality of life advantage from the use of cabozantinib compared to the previous standard of care, sunitinib. Sunitinib had been the main VEGF TKI used since its FDA approval in 2006 [11], hence, the *de facto* comparator arm for all the contemporary first-line clinical trials for advanced and metastatic RCC. While VEGF TKI monotherapy is fast shrinking and narrowing as a treatment option for first-line therapy in metastatic RCC, the Checkmate 214 trial shows that patients with favorable risk disease per the International Metastatic Renal Cell Carcinoma Database Consortium (IMDC) criteria tend to still do better with sunitinib monotherapy compared to IO combination therapy. Therefore, extrapolation has been made to the ALLIANCE-led CABOSUN trial, which while technically enrolled 157 patients with intermediate-risk and poor-risk disease only and randomized to either cabozantinib or sunitinib, the FDA approval and label encompasses all patients in first-line, without distinction regarding their risk of disease, given that the primary endpoint of progression-free survival was met at 8.6 months (95% confidence interval [CI] 6.8—14.0) for cabozantinib compared to sunitinib at 5.3 months (95% CI 3.0—8.2) at a hazard ratio [HR] 0.48 [95% CI 0.31—0.74] [8, 12, 13].

The ability to tolerate side-effects of VEGF TKIs are important, whether used as monotherapy or in combination with CPI. Different measures of quality of life (QOL) analyses have been attempted, and the recent analyses [10] as well as interpretation of this data [14], offered some insights as to the evaluation of the impact of QOL and tolerance to these drugs.

The Q-TWiST measures have been used for in clinical trials to add perspective and assess toxicities of therapy as it relates to the clinical parameters of benefit such as PFS [15]. Just as important to grading the toxicity is evaluating patients’ perceptions regarding clinical symptoms [16], but ultimately to comparing to current standards of care [17], as well as formally using different gauges of response as well as toxicity to dictate the value and decrements in quality of life [18].

The Q-TWIST analyses for the CABOSUN trial [10] is but one analysis of such quality of life parameters which looked at the time without symptoms of disease or toxicity defined as the “TWiST”, the “TOX” or the time without disease progression and without any development of toxicity as well as the “REL” or relapse, time after disease progression until death. The parameters were measured as utility scores which ranged from 0 to 1, with a point scale of 1 referring to one’s perfect health while a score of 0 equating to death. Not surprisingly, the CABOSUN trial reflected statistically significant difference within all parameters that was in favor of cabozantinib over sunitinib, despite both drugs being VEGF TKIs. Different VEGF TKIs are also expected to yield different toxicity findings. Further analyses of toxicity were extrapolated from the METEOR trial [19], which is a second-line trial assessing the difference between cabozantinib and everolimus post-TKI treatment. Other Q-TWiST analyses from different trials such as the comparison between sunitinib and pazopanib showed favorable results for pazopanib [20]. However, VEGF TKI monotherapy would now be relevant for use only in a relatively small population of metastatic RCC patients, typically those with favorable risk disease per IMDC criteria, who would be appropriate for VEGF TKI monotherapy alone. Majority of patients would be otherwise suitable for at least an IO/IO combination or an IO/TKI combination therapy if they have at least intermediate-risk or poor-risk disease. Therefore, emphasizing QOL analyses for the more contemporary trials would be of relevant importance such as the one in the Checkmate 214 trial [2], with the Q-TWiST analyses of nivolumab and ipilimumab showing improvement over sunitinib with 3.5 months with a relative gain of 15.1% compared to sunitinib [21]. There has been reported Q-TWiST analyses for other second-line therapy trials such as the comparison between nivolumab and everolimus in the CheckMate 025 trial in favor of nivolumab [22] and the phase III trial on temsirolimus over interferon [23] or sunitinib over interferon [24], all of which closely followed the primary endpoint results of the corresponding trials as well. Beyond Q-TWiST analyses, it would also be helpful to evaluate patient reported outcomes (PROs) as additional gauge for understanding patients’ perception and tolerance of these regimens. For instance, the Checkmate 214 trial showed that treatment with nivolumab and ipilimumab showed less deterioration of symptoms and health-related quality of life overall compared to the standard of care arm, sunitinib [25].

It would be interesting to note how the newer VEGF TKIs that have just been approved such as tivozanib [26], would be placed in sequencing for third line therapy and beyond, along with QOL analyses. While there will unlikely be data to inform the most optimal sequencing approach after failure from prior VEGF TKI/IO combination therapy or IO/IO therapy, the subsequent treatment approach may hinge upon the toxicities incurred from first-line therapy as well as duration or durability of response from first-line therapy. In addition, further evaluation and QOL analyses for the appropriate second-line therapy would also be helpful to guide future therapy.
